# Role of mitophagy in the hallmarks of aging

**DOI:** 10.7555/JBR.36.20220045

**Published:** 2022-08-28

**Authors:** Jie Wen, Tingyu Pan, Hongyan Li, Haixia Fan, Jinhua Liu, Zhiyou Cai, Bin Zhao

**Affiliations:** 1 Department and Institute of Neurology, Guangdong Medical University, Zhanjiang, Guangdong 524001, China; 2 Guangdong Key Laboratory of Aging-related Cardiac and Cerebral Diseases, Zhanjiang, Guangdong 524001, China; 3 Chongqing Key Laboratory of Neurodegenerative Diseases, Chongqing 400013, China; 4 Department of Neurology, Chongqing General Hospital, Chongqing 400013, China; 5 Department of Neurology, the Affiliated Hospital of Southwest Medical University, Luzhou, Sichuan 646000, China; 6 Chongqing Medical University, Chongqing 400042, China

**Keywords:** mitophagy, aging, dietary restriction, telomere shortening, epigenetic alterations, protein imbalance

## Abstract

Aging, subjected to scientific scrutiny, is extensively defined as a time-dependent decline in functions that involves the majority of organisms. The time-dependent accretion of cellular lesions is generally a universal trigger of aging, while mitochondrial dysfunction is a sign of aging. Dysfunctional mitochondria are identified and removed by mitophagy, a selective form of macroautophagy. Increased mitochondrial damage resulting from reduced biogenesis and clearance may promote the aging process. The primary purpose of this paper is to illustrate in detail the effects of mitophagy on aging and emphasize the associations between mitophagy and other signs of aging, including dietary restriction, telomere shortening, epigenetic alterations, and protein imbalance. The evidence regarding the effects of these elements on aging is still limited. And although the understanding of relationship between mitophagy and aging has been long-awaited, to analyze details of such a relationship remains the main challenge in aging studies.

## Introduction

Aging is a complex multifactorial biological process that affects virtually all cells and tissues in the body, leading to impaired functions and loss of homeostasis^[1]^. There is evidence that genetic damage accumulates in somatic tissues during mammalian aging^[2–3]^. Indeed, age-related increases in reactive oxygen species (ROS) are considered to be responsible for the aging process^[4]^, while mitochondrial dysfunction is deemed to be a hallmark of aging^[5]^.

Mitochondria, double membrane-bound organelles in the organism, have several functions in a eukaryotic cell^[6–7]^. Mitochondria quality monitoring is an integrated system that alters mitochondrial structure and coordinates multiple processes such as mitochondrial fission, fusion, biogenesis, bioenergetics, protein stabilization, and degradation through mitophagy^[8]^ (see Mitophagy section). Current evidence overwhelmingly suggests that various features of human brain aging are closely associated with mitochondrial dysfunction at the cellular level^[9]^, leading to oxidative stress and biological energy deficiency in multiple neural cells in neural tissue that are extremely sensitive to energy deprivation. Adenosine triphosphate (ATP) is generated by oxidative phosphorylation of the respiratory chain in mitochondria and is responsible for providing power for nearly all cellular activities, and the simultaneous generation of ROS can be detrimental. It may result in severe impairment of mitochondrial proteins, DNA, and lipids^[10–11]^, thus contributing to aging and age-related diseases in an unavoidable but random process^[12]^. It will cause oxidative stress and cell death, if the excessive production of ROS is higher than the cell's antioxidant capacity. Mitochondrial dysfunction results in higher ROS production and promotes the programmed cell death^[10]^. With aging, intrinsic cellular damage can affect proliferating cells, resulting in cell senescence, while the ability of macrophages to clear senescent cells decreases, for example, as a result of a diminished immune response^[13]^. Beyond that, mitochondrial physiology may have a powerful link to human aging. Existing evidence suggests that mitochondria may play an essential part in the inflammatory phenotype^[14]^. Activation of circulating neutrophils by the release of mitochondrial DNA (mtDNA) and formyl polypeptides mediates tissue injury^[15]^. Damage-associated molecular patterns and related pathogen-associated molecular patterns can provoke the assembly of inflammasomes or high-molecular-weight protein complexes that function to initiate caspase-1^[16]^. Importantly, activation of caspase-1 can cause mitochondrial impairment^[17]^. There may be a vicious cycle between mitochondrial damage and aging.

Alzheimer's disease (AD) brain showed abnormal accumulation of mitochondria with ultrastructural changes, such as shrinkage and intimal crest rupture^[18–19]^. Mitochondrial abnormalities may contribute to AD. Dynamin-related protein 1 (DRP1) belongs to a large family of evolutionarily conserved large GTPases. DRP1 is involved in several fundamental aspects of mitochondria and is related to various cellular functions^[20]^. In a transgenic tau mouse model of AD, partial downregulation of DRP1 alleviates cognitive behavior and enhances mitophagy, autophagy, and dendritic spines^[21]^. Synaptic damage and mitochondrial dysfunction were early events in AD pathogenesis^[22]^. In conclusion, a tight control of the number and activity of mitochondria is required to adapt the cells to the metabolic energy state and remove abnormal functioning mitochondria on time. Currently, there are several articles summarizing the role of mitochondria in neurodegenerative diseases, but there are few articles on mitophagy in this area. This paper reviews the tight link between mitophagy and aging mechanisms in several aspects to provide a few potential insights into anti-aging and prevention of aging-related diseases.

## Mitophagy

**Figure 1:**
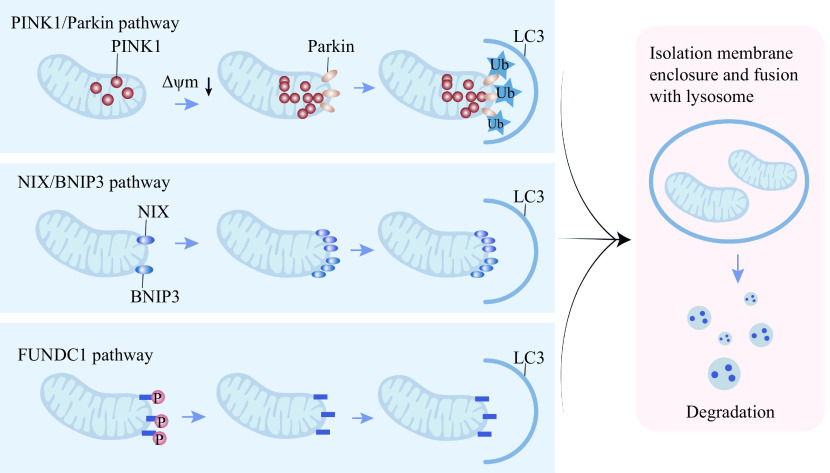
Mitophagy.

Mitochondria were first identified in autophagosomes in 1957, and autophagy was hypothesized to play a vital part in mitochondrial removal. Mitochondrial selective autophagy removal, referred to as mitophagy, is strongly associated with prevention standard quality control procedures in certain types of human pathobiology, particularly in neurodegeneration such as Parkinson's disease and Wolfram's syndrome 2^[7,23–24]^. Mitophagy in neurons is achieved by two mechanisms, *i.e.*, nonreceptor-mediated mitophagy and receptor-mediated mitophagy^[25–26]^ (***Fig. 1***): (1) Non-receptor-mediated mitophagy or classical mitophagy involves phosphatase and tensin homolog-induced kinase 1 (PINK1) and Parkin. PINK1 is typically degraded by a presenilin-associated rhomboid-like protease in the mitochondrial inner membrane^[27–28]^. PINK1 becomes active during mitophagy induction and accumulates on the outer mitochondrial membrane (OMM), where Parkin and ubiquitin are recruited *via* phosphorylation to trigger proteasome degradation^[7,29–31]^. Moreover, ubiquitination of Parkin by OMM proteins results in enhancing PINK1 protein activity and substrate phosphorylation^[30,32]^. (2) Mitophagy is also promoted by specific mitochondrial junction proteins, including BCL2/adenovirus E1B 19 kDa protein-interacting protein 3 (BNIP3)^[33]^, BNIP3-like protein (BNIP3L, also known as NIP3-like protein X [NIX])^[34]^, and FUN14 domain-containing protein 1 (FUNDC1)^[35]^. BNIP3 and NIX are positioned in the OMM and are members of the BH3-only structural domain protein of the BCL2 family, which plays an essential role in regulating BAX/BCL2-associated apoptosis^[34]^. Receptor-mediated mitophagy, initiated by mitochondrial receptor proteins containing microtubule-associated protein 1A/1B-light chain 3 (LC3)-interacting region (LIR) motif (W/F/YxxL/I), is activated under certain circumstances^[36–37]^. For example, during hypoxia, transcription of BNIP3 and NIX is triggered by hypoxia-inducible factor 1α^[38]^. The activity of BNIP3 and NIX is modulated by phosphorylation, and an increased phosphorylation contributes to their binding affinity for LC3^[39–40]^. Hypoxia also enhances the binding of FUNDC1 to LC3 through phosphoglycerate mutase family member 5-mediated dephosphorylation^[35,41–42]^. In contrast, ULK1-mediated phosphorylation of FUNDC1 is also a mitophagy activating event. Eventually, ubiquitination of FUNDC1 by E3 ubiquitin-protein ligase 5 facilitates lysosomal degradation in mitochondria^[35,43]^. Mitophagy induced by FUNDC1 is shown to reverse mitochondrial membrane potential, diminish mitochondrial ROS production and restrain mitochondria-induced apoptosis^[44–45]^. In addition, there is an interaction between two mitophagy pathways mentioned above, in which BNIP3 interacts with PINK1 to suppress its clearance and promote Parkin recruitment and PINK1/Parkin-mediated activation of mitophagy^[46]^.

Transcellular mitophagy occurs when brain nerve cells deliver mitochondria at the synapse, and these extracellular mitochondria are eaten by microglia^[47–48]^. Previous research revealed that in mice mitochondria were shed by retinal ganglion axons and then cleaved by neighboring astrocytes^[48]^. Mitochondria may undergo fragmentation in the axoplasm with the cooperation of axon lysosomes^[47]^. Instead of being degraded within the somatic cell, axonal mitochondria are surrounded by the axoplasmic membrane, which is shed and degraded by the surrounding cells^[26]^. Understanding how mitophagy is altered during aging can help identify meaningful interventions to slow down aging and enhance age-related health outcomes.

Mitophagy limits the generation of ROS, blocks the accrual of mtDNA mutations, increases ATP formation, and prevents apoptotic signaling and the activation of inflammatory vesicles^[49–50]^. The contribution of defective mitophagy to aging and age-related diseases highlights the importance of controlling mitochondrial quality and quantity through mitophagy. Mitophagy injury results in the gradual accumulation of defective organelles, mainly damaged mitochondria that are sources of oxidative stress in cells. In AD, elevated levels of amyloid-beta and p-tau induce ROS production, leading to excessive mitochondrial fission and promoting defective mitophagy^[51]^. In the brains of AD patients, the mitochondrial abnormalities include age-dependent accumulation of mtDNA, decreased mitochondrial membrane potential, enhanced ROS generation, decreased mitochondrial axonal transport, reduced mitochondrial ATP, decreased mitochondrial enzyme activity, and increased mitochondrial fragmentation^[52–54]^. The mitochondrial abnormalities lead to defective mitophagy in AD patients^[51]^.

Mitophagy is a critical process for maintaining cellular health. It facilitates mitochondrial turnover and protects against the accumulation of dysfunctional mitochondria that may cause cellular degeneration. In addition to selective clearance of damaged mitochondria, mitophagy requires adjusting mitochondrial numbers to accommodate changes in cellular metabolic demands to achieve steady-state mitochondrial turnover^[55–58]^. Thus, mitochondrial and mitophagy interact with each other to achieve these goals.

## Mitophagy and the hallmarks of aging

### Mitophagy and dietary restriction

**Figure 2:**
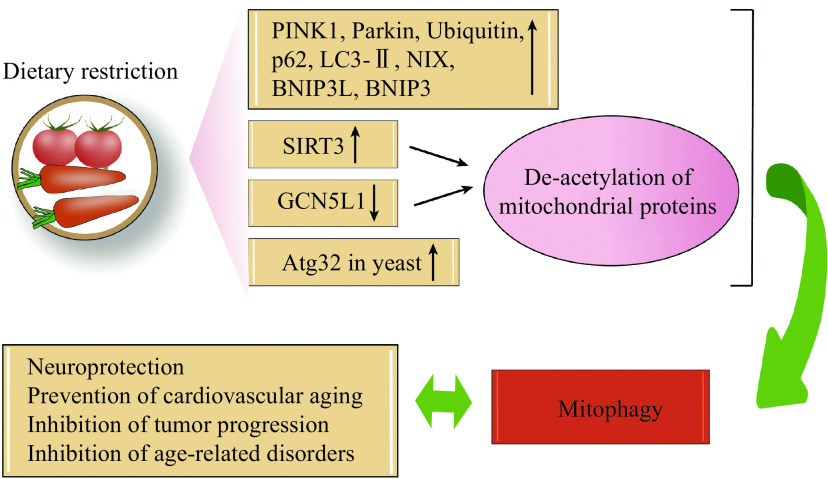
Mitophagy and dietary restriction.

Dietary restriction (DR), also known as calorie restriction (CR), is a moderate decrease in overall food intake without nutritional deficiencies. DR is also used to characterize dietary interventions that involve a reduction or weakness in particular nutritional components^[59–61]^. Accumulating evidence suggests that DR, including the changes in calorie intake, diet composition, or timing of food intake, has a vital part in the prevention or treatment of chronic disease^[62]^. Among the various stimuli that trigger mitophagy, DR is one of the most potent non-genetic triggers that initiate the process of mitophagy. The main effects of DR on lifespan are managed by interacting pathways. In the case of sufficiency of nutrients, the nutrient-sensing signaling cascades are activated to promote cell growth, leading to biological development and proliferation. Significant effects of DR on lifespan and health are regulated by overlapping pathways. In the presence of an adequate nutrient supply, nutrient-sensing signaling cascades are activated to promote cell growth, leading to biological maturation and proliferation^[62–67]^. The majority of studies have shown that fasting or CR induces mitophagy and markers associated with mitophagy, such as BINP3 and Parkin (***Fig. 2***). There are two mechanisms to reduce the accumulation of damaged proteins *via* CR: (1) the short-term starvation could lead to an increased protein hydrolysis and reduce the accumulation of damaged proteins^[68]^, in which CR is responsible for the reduced protein turnover and the reduced need for protein replacement^[69]^; (2) the data suggest that CR reduces oxidative damage, maintains oxidative capacity, and activates peroxisome proliferator-activated receptor-γ coactivator 1alpha (PGC-1α), indicating an upregulation of mitochondrial biogenesis^[70–72]^.

The mammalian growth axis is composed of growth hormones produced by the anterior pituitary, and its secondary mediator, insulin-like growth factor 1 (IGF-1), produced by many cell types in response to growth hormones. The IGF-1 receptor-dependent pathway is inhibited by CR, which decreases nuclear factor-kB activity and reduces the pro-inflammatory effects of aging^[73]^. The intracellular signaling pathway of IGF-1, which alerts cells to the presence of glucose, is the same as the insulin-induced signaling pathway, both referred to as the "insulin and IGF-1 signaling" (IIS) pathway. Notably, the IIS pathway is the most evolutionarily conserved pathway for the control of aging, and its multiple targets include the FOXO transcription factor family and the mammalian target of rapamycin (mTOR) complex, which are also involved in the aging process and remain conserved during evolution^[74–75]^. In humans and model organisms, genetic polymorphisms or mutations, which may decrease the functions of intracellular downstream effectors, such as GH, IGF-1 receptor, insulin receptor or AKT, mTOR, and FOXO, are linked to longevity, further suggesting a significant role of nutritional and bioenergetic pathways in prolonging lifespan^[74–75]^.

In parallel to the IIS pathway involved in glucose sensing, three other trophic sensing systems are (1) mTOR, the primary focus of ongoing research, for sensing high amino acid concentrations; (2) AMPK, for sensing low energy states by measuring higher AMP levels; and (3) sirtuins, sensing low energy states by measuring high NAD^+^ levels^[75]^.

The mTOR kinases are part of two multiprotein complexes, mTORC1 and mTORC2, which regulate various aspects of anabolism^[76]^. Genetic downregulation of the mTORC1 activity in yeast, worms, and flies prolongs lifespan and attenuates the longevity benefits of DR, which suggests that mTOR suppresses the DR phenotype^[77]^. DR decreases the activity of mTORC1 in invertebrates and several mammals. In yeast, DR does not further prolong replication lifespan when deficiency of genes encoding mTOR and ribosomal protein S6 kinases (S6K) homologs occurs^[78]^. However, the interaction between DR and mTORC1 signaling is complex. It has been reported that combined with dietary restriction in nematodes, RNAi knockdown of S6K and translation initiation factor results in a prolonged lifespan^[79]^.

The two additional nutrient sensors, AMPK, and sirtuins, act in the opposite direction to IIS and mTOR, pointing to nutrient scarcity and catabolism rather than nutrient enrichment and anabolism^[80]^. In the absence of AMP-dependent kinase and fibroblast growth factor 21 genes, mitophagy in muscle is reduced, under both fasted and fed conditions^[69]^. Thus, their upregulation facilitates non-pathological aging. Activation of AMPK has diverse impacts on metabolism and significantly shuts down mTORC1^[80]^.

Collectively, the influence of diet on mitophagy is complex. There is no doubt that DR triggers mitophagy, but the effect of nutritional enrichment on mitophagy needs to be further studied, although potential evidence of this effect already exists.

### Mitophagy and telomere

Telomeres, with unique structures, safeguard the end of linear chromosomes, and secure the stability of the genome from nuclear fission degradation, unwanted recombination or repair, and interchromosomal fusion. Telomeres consist of a long train of repetitive G-rich DNA that is bound together by a specialized six-subunit protein complex, the shelter, and telosome complex. In somatic cells, protective proteins not only protect telomeres from DNA damage responses but also regulate telomere length^[81]^. A small fraction of telomeric DNA is lost as cells divide, and telomeres become shorter with age^[82]^. Telomere length, consequently, acts as a biological clock that determines the lifespan of cells and organisms. The majority of DNA polymerases cannot fully replicate the end of linear DNA, and telomerase is the only specialized form capable of doing so. Telomerase, a ribonucleoprotein complex consisting of reverse transcriptase and non-coding RNA, can prevent telomere shortening by adding DNA sequence repeats (TTAGGG) to the 3′ end of the DNA strand in the terminal telomeric region of eukaryotic chromosomes^[83]^. However, in most mammalian cells, telomerase is not expressed, eventually causing the progressive loss of telomere protection factors^[84]^. Telomere shortening limits the ability of cells to proliferate, and telomere reduction causes cellular senescence, thereby impeding cell cycle progression and halting replication. The number of senescent cells increases with age. Cellular senescence is a sequence of cellular states when cells undergo phenotypic changes, after their initial growth has ceased, and thus aging includes these two diverse events; aging is a gradual decline over time, but senescence appears to occur throughout the lifespan, which also contains embryogenesis. These events are not only related to autophagy but also directly related to mitophagy^[85]^.

**Figure 3:**
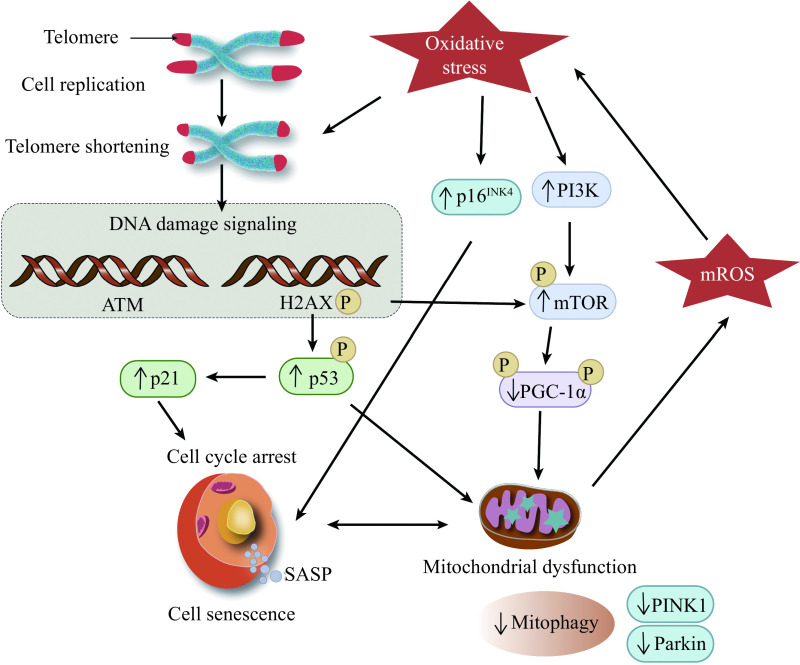
Mitophagy and telomere.

Hayflick and Muirhead first described senescence as an irreversible growth arrest associated with telomere wear^[86]^. When telomere length meets a limiting threshold, the cell suffers senescence and/or apoptosis. However, aging may be a beneficial compensatory response that helps prevent the continuous replication of damaged cells and removes tissue-damaged and potentially cancer-causing cells. The death of damaged cells is triggered by the immune system^[13]^, and functions in embryonic development, wound healing, tissue repair, and aging. With cell aging, new cells replace old ones and, ultimately, the regenerative potential of the tissue diminishes as the stem cells are depleted. Repeated cell division causes continuous shortening of telomeres, activating the DNA damage response and causing activation of p53 (***Fig. 3***), and arresting cell cycle or initiating cellular senescence. Telomere shortening also activates the p53 and mTOR signaling pathways, thereby inhibiting PGC-1α, a transcription factor that is a vital regulator of mitochondrial function^[87–89]^. Abnormal mitochondrial functioning produces mitochondrial reactive oxygen species (mROS) that probably further damage telomeres and promote senescence.

In fact, studies found that aged mice had a reduction of cell cycle activity and division of hematopoietic stem cells compared to young mice^[90]^. These reduced divisions are associated with the accumulation of DNA lesions, leading to overexpression of cell cycle inhibitory proteins (*e.g*., p16^INK4a^), a known promoter of cellular senescence. With cell aging, DNA lesions accumulate not only in chromosomal DNA but also in mitochondrial DNA, contributing to mitochondrial dysfunction, which may be responsible for electron leakage and energy deficiency as mitochondria play an essential part in the respiratory oxidation process of ATP production^[91]^. There are relatively few studies on the correlation between telomeres and mitophagy; nevertheless, mitochondria may be expected to be used as a starting point to study the correlation.

### Mitophagy and epigenetic alterations

**Figure 4:**
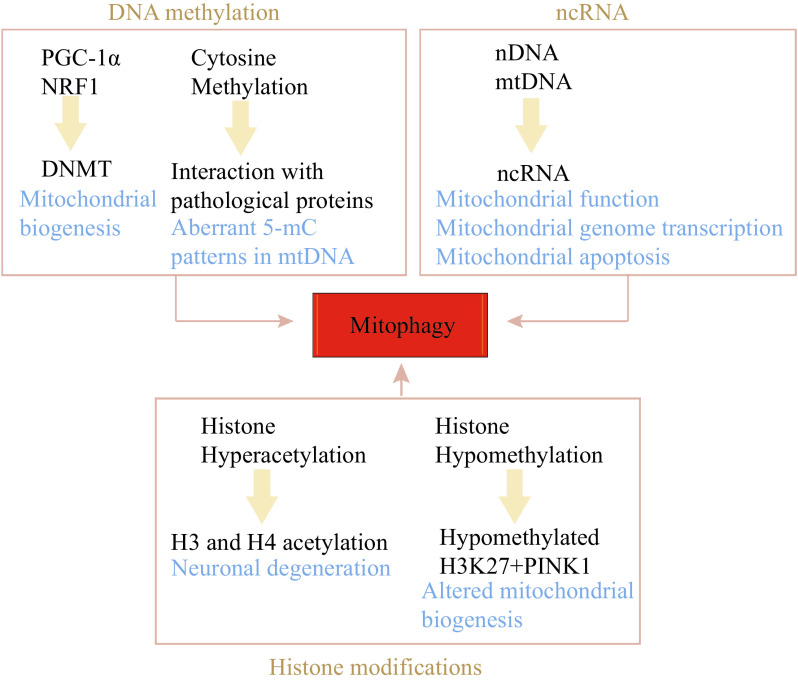
Mitophagy and epigenetics.

Different cells within an organism have identical DNA with the same nucleotide sequences. However, they produce various gene products, and the phenomenon that controls this mechanism is called epigenetics. These epigenetic substances have been documented to be involved many pathological processes, including dysfunctional mitochondria^[92]^. Epigenetic changes are associated with altered DNA methylation patterns, post-translational modifications of histones, chromatin remodeling, and non-coding RNAs (ncRNAs) (***Fig. 4***), all of which can alter protein expression^[93]^. Epigenetic modifications may be a manifestation of genetic alterations that can signify aging. The increases in histone H4 at lysine 16 (H4K16) acetylation, H4K20 trimethylation, or H3K4 trimethylation, and decreases in H3K9 methylation or H3K27 trimethylation comprise age-associated epigenetic hallmarks^[93–94]^.

#### DNA methylation

Methylation patterns are instituted in the developing embryo by two DNA methyltransferases, DNA (cytosine 5) methyltransferase 3a (DNMT3a) and DNA (cytosine 5) methyltransferase 3b (DNMT3b)^[95]^. DNMT1 is the sole member of the three known catalytically active DNA methyltransferases that target mitochondria. Mitochondrial DNMT1 (mtDNMT1) combines with mtDNA, demonstrating the existence of mtDNMT1 in the mitochondrial matrix. Mitochondrial epigenetics is regulated by mtDNMT1 variants upregulating DNMT1 initiation sites *via* hypoxia-dependent factors such as PGC-1α, nuclear respiratory factor 1 (NRF-1) released from mitochondria through p53^[96]^. In the nucleus, cytosine methylation collaborates with N-terminal histone modifications to build silent chromatin structures, which in turn control the performance of nuclear genes^[97]^. Significant levels of 5-hydroxymethylcytosine were found in the DNA of neurons, brain^[98]^, and embryonic stem cells^[99]^. DNMT3b deletion also regulates mitochondrial dynamics, upsets fission and fusion, decreases mitochondrial DNA levels, and shifts the paradigm from glycolysis to oxidative phosphorylation. In addition, one study has showed that DNMT1 preserves the metabolic health of adipocytes and serves as an epigenetic safeguard for mitochondrial dynamics^[100]^. So far, it has not been directly experimentally demonstrated, however, that the lifespan of an organism can be extended by varying the pattern of DNA methylation^[13]^.

#### Histone modifications

Histone modifications, such as acetylation and demethylation, are another epigenetic feature that has contributed significantly to mitochondrial epigenomics. The absence of histone methylation complex (H3K4 and H3K27) components prolongs the lifespan of nematodes and Drosophila, respectively^[101–102]^. Genome-wide research has detected that acetylation is associated with the availability of acetyl-CoA. Since a majority of acetyl-CoA is produced in the mitochondrial matrix^[103]^, studies have demonstarted that cells are sensing nutritional signals by perceiving the effectiveness of acetyl-CoA and integrating it into epigenetic mechanisms^[104]^. In addition, histone demethylases cause histone demethylation and demand many mitochondria-dependent metabolic auxiliaries, indicating their engagement in mitochondrial regulation^[105]^.

Mitochondria contain genomes and generate mitochondria-specific nucleic acids and proteins. Powerful interactions between mitochondria and the nucleus are required to be mediated by encoded ncRNAs and proteins to maintain the homeostasis throughout the cell. Long non-coding RNAs (lncRNAs), longer than 200 bp in length, are engaged in the modulation of nearly every step of gene expression and are involved in a broad range of diseases, such as cancer^[106]^. They play an essential part in the immune regulatory system^[107]^. In terms of coordinating signaling systems, part of lncRNA is transcribed in the nucleus and plays a pivotal part in regulating mitochondrial functions or dynamics. A recent study has showed that lncRNA maternally expressed gene 3 facilitates FUNDC1-related mitophagy, thereby diminishing mitochondrial apoptosis and alleviating cognitive impairment in diabetes^[106]^. LncRNAs can modulate a multitude of cellular functions to meet the needs of tumor metabolism, including mitochondrial metabolic reprogramming. New knowledge using the lncRNA-mitophagy-cancer axis may offer potential new targets for human anti-aging therapy^[108–109]^.

Mitochondria must coordinate precisely with the nuclear genome to assure appropriate cellular function and energy homeostasis^[110–111]^. Recent studies have demonstrated that, in mitochondrial-nuclear crosstalk, lncRNAs may serve as novel retrograde and cis-acting signaling molecules^[108]^. The lncRNA is encoded not only by the nuclear genome but also by the mtDNA. The mitochondrial genome encodes 13 mRNAs, 22 tRNAs, and two rRNAs, which produce proteins involved in oxidative phosphorylation. In a further step, there are LncND5, LncND6, and LncCyt B, whose manifestations are cell and tissue-specific, indicating that they play an instrumental role in the regulation of mitochondrial gene expression^[112]^. At least 18 mitochondria-related ncRNAs have been detected, which play significant roles in manipulating mitochondrial functions, modifying metabolic reprogramming, mitochondrial genome transcription, stress signaling, and mitochondria-related apoptosis^[108]^. Although the mechanism by which lncRNAs regulate mitophagy remains unclear, the nuclear genome-encoded lncRNA metastasis-associated lung adenocarcinoma transcript 1 (MALAT1) regulates mitophagy by shuttling to the mitochondria and acting as a novel epigenetic messenger^[108]^. Mitophagy events were significantly reduced after the knockdown of MALAT1; as observed in MALAT1-deficient hepatocellular carcinoma cells, mitophagy proteins, notably PINK1, p62, NDP52, BNIP3, and LC3, were decreased in regulation^[113]^. Although the specific molecular mechanisms in which the mitophagy process affects neurodegeneration remain unclear, there is a growing evidence that ncRNAs, various endogenous regulators, including microRNAs and lncRNAs, are extensively involved in the mitophagy process and play critical roles in the aging process and neurodegenerative diseases^[114]^.

In recent years, epigenetics has been studied extensively by the scientific community. Overall, mastering and manipulating the epigenome has shown some promise for preventing age-related disease development. The relationship between epigenetics and mitophagy is also urgent to be further explored.

### Loss of proteostasis

Intracellular proteins are highly active molecules that undergo intense conformational alteration and partial, or total unfolding/refolding of transmembrane translocation, and finally assemble into functional structures or regulate the function of themselves and other interacting proteins^[115]^. Proteostasis relies on multiple molecular mechanisms to regulate protein folding, ranging from adaptation for protein synthesis and degradation, to promotion for the course of post-translational protein folding and maturation^[116]^. Aging is also associated with the aggregation of mtDNA and protein damage^[117]^. All cells use a range of quality control mechanisms to maintain the integrity and functions within their proteome. Protease homeostasis concerns the mechanism of stabilization of the correctly folded proteins, most notably the heat-shock family of proteins and the proteasomal or lysosomal mechanism of protein degradation^[115,118–119]^. All such systems work in a harmonized manner to revive misfolded peptide structures or to eliminate and degrade these entirely, thereby preventing the aggregation of impaired elements and ensuring the constant updating of cellular proteins^[115]^. Furthermore, there is a chronic expression of non-folded, misfolded, or assembled proteins that promotes the development of certain age-related diseases, such as AD and Parkinson's disease^[120]^.

**Figure 5:**
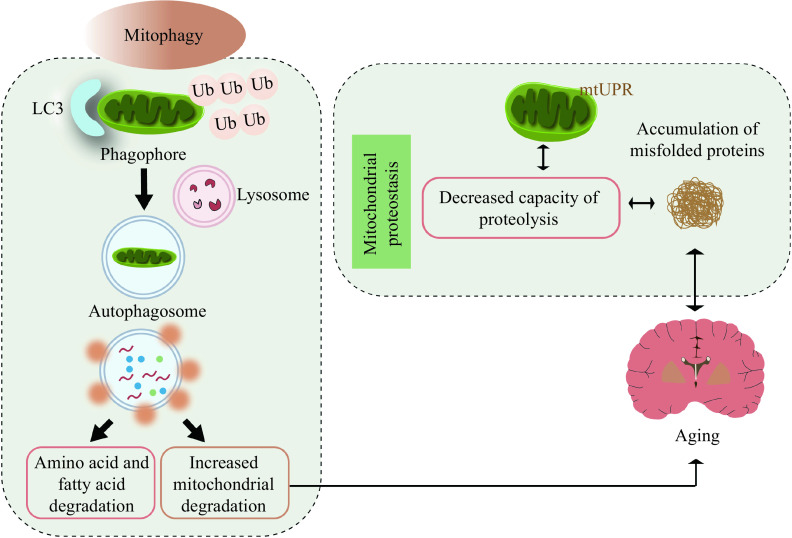
Mitophagy and loss of proteostasis.

Mechanisms of mitophagy have been investigated in studies of yeast, and several proteins are essential for mitophagy. Kissova *et al* has reported the first evidence that mitophagy in yeast is controlled by genes. They have revealed that Uth1p, a member of the so-called SUN family, is essential for mitophagy aiming at the identification of proteins involved in the regulation of yeast lifespan^[121]^. Atgs are exploited in all autophagic pathways, and several have been known to work in selective mitochondrial uptake. Being analogous to Uth1p, Atg32 is required for mitophagy, but not for nonspecific autophagy. Atg32 is able to bind to Atg11 and interacts with Atg8, which is considered the signal for mitochondrial uptake into autophagosomes^[122–123]^. These proteins are integral to mitophagy in yeast. However, the pathway by which they initiate mitophagy on their own in mammalian cells remains to be discovered. Mitochondria have a crucial role in defending cells from oxidative stress, but they are not protected against the damaging role of reactive species on their own. A study revealed that damaged mitochondria might be sufficient to trigger mitophagy^[124]^. Mitochondrial dynamics are altered under stressful conditions (***Fig. 5***). Mitochondrial unfolding protein response (mtUPR) is a stress response that enhances the expression of mitochondrial proteases and chaperone proteins to relieve mitochondrial stress arising from mistranslated and/or misfolded proteins and to promote biogenesis to preserve protease stability and mitochondrial function^[125]^. The activity of two major protein hydrolysis systems involved in protein quality control, the autophagy-lysosome system and the ubiquitin-proteasome system, decreases as one age^[126]^, supporting the view that the breakdown of protein homeostasis is a shared feature of the elderly^[13]^.

The regulators of mitochondrial biogenesis are the PGC-1α transcriptional family, and these regulators interact with NRF-1 and NRF-2 to modulate protein expression. The production of new mitochondria is under the coordinated control of multiple pathways, among which PGC-1α facilitates the interaction with the nuclear receptor family through a specific LXXLL (where L is leucine and X is any amino acid) recognition domain, resulting in an improved transcription^[127]^. Mitochondria provide their translation and protein quality control mechanisms in the cell, including chaperones and proteases within the mitochondrial matrix, maintaining mitochondrial protein homeostasis^[128]^. These findings suggest that impaired/dysfunctional mitochondrial catabolism through mitophagy during aging is the leading cause of loss of protease homeostasis in skeletal muscle^[129–130]^. During normal physiological aging, both mitochondrial functions and the capability of cells to undergo mitochondrial autophagy are expected to decline; these impacts are exacerbated in diseases associated with aging, such as AD, which would accelerate the decline of mitochondrial functions^[85]^.

## Conclusions and perspectives

In this review, we present a close relationship among mitophagy and dietary restriction, telomere deletion, protein imbalance, epigenetic alterations, and direct or indirect effects of these relationships on aging. Among the protein imbalances, epigenetic alterations, and telomere deletions, the common hallmark of these features is undoubtedly hostile and associated with DNA damage. Optimal nutrition sensing is essential for existence, but in excess, it may turn out to be pathological. Mitophagy may also interact with senescent cells and stem cells, and information exchange between these cells influences aging. These features may be synergistic or antagonistic, becoming progressively negative in a process partially facilitated or accelerated by the main part. Ultimately, integrative hallmarks emerge, when the accumulated damage induced by significant and opposing profiles fail to be compensated by the self-balancing mechanisms within the tissue.

Mitochondria are critical for cell survival and death, which means that mitochondrial homeostasis in response to cellular stress must be strictly controlled. Mitophagy is such a fundamental mechanism for mitochondrial quality and quantity control. Several studies have illustrated that there is a large amount of intercommunication among multiple mitophagy pathways, which can coordinate and complement each other to function in various organs of the body. The role of PINK1 and Parkin in cardiac functions has been widely reported. In terminal heart failure patients, PINK1 levels are significantly reduced. PINK1-deficient mice suffer from left ventricular insufficiency and pathological myocardial hypertrophy featuring increased oxidative stress and compromised mitochondrial functions^[131]^. Unlike PINK1, mice deficient in Parkin are susceptible to cardiac infarction, thereby reducing their survival^[132]^. Intriguingly, mtDNA released from compromised mitochondria provokes an inflammatory response in heart muscle cells, ultimately leading to cardio-inflammation and expansive cardiomyopathy^[133]^. Aging contributes to synapses, autophagy, and mitophagy in AD progression and pathogenesis^[85]^. Synaptic lesions and impaired mitophagy are early signs of progression of the disease, which may provide some new insights into human lifespan in terms of delaying aging.

The accelerated evolution of next-generation sequencing technologies might make a particular contribution to aging investigations, helping to assess the specific accumulated inheritance and epigenetic variations in individual cells during aging^[134]^. Mitophagy may be a promising target, pioneering new pathways for the treatment of neurodegenerative diseases. Still, more research is required to define the role of mitochondria in the process of aging. Further research may result in the development of therapies based on mitochondrial phagocytosis to slower or even reverse the adverse effects of aging.
